# Foodborne Outbreak and Nonmotile *Salmonella enterica* Variant, France

**DOI:** 10.3201/eid1801.110450

**Published:** 2012-01

**Authors:** Simon Le Hello, Anne Brisabois, Marie Accou-Demartin, Adeline Josse, Muriel Marault, Sylvie Francart, Nathalie Jourdan Da Silva, François-Xavier Weill

**Affiliations:** Institut Pasteur, Paris, France (S. Le Hello, M. Accou-Demartin, A. Josse, F.-X. Weill);; Agence Nationale de Sécurité Sanitaire de l’Alimentation, de l’Environnement, et du Travail, Maison-Alfort, France (A. Brisabois, M. Marault);; Ministère de l’Alimentation de l’Agriculture et de la Pêche, Paris (S. Francart);; Institut National de Veille Sanitaire, Saint-Maurice, France (N. Jourdan-Da Silva)

**Keywords:** bacteria, foodborne infections, outbreak, nonmotile Salmonella, Salmonella enterica, food poisoning, laying hens, poultry, eggs, tiramisu, France

## Abstract

We report a food-related outbreak of salmonellosis in humans caused by a nonmotile variant of *Salmonella enterica* serotype Typhimurium in France in 2009. This nonmotile variant had been circulating in laying hens but was not considered as Typhimurium and consequently escaped European poultry flock regulations.

Identification of *Salmonella enterica* serotypes is based on flagellar and somatic antigens. Of the 2,610 serotypes distinguished in the Kauffmann-White-Le Minor Scheme (*1**,**2*), only Gallinarum are obligatory nonflagellate and hence, nonmotile. Nonmotile variants of diphasic *Salmonella* spp. are rarely reported in humans, food, or animals (*3**,**4*) as opposed to monophasic variants (*5*), in particular the monophasic variant of serotype Typhimurium (antigenic formula 1,4,[5],12:i:–) that has emerged worldwide (*6**–**8*). The French National Reference Center for *Salmonella* (FNRC-Salm) network is based upon voluntary reporting from ≈1,500 hospital and private clinical laboratories representing two thirds of all clinical *Salmonella* spp. strains isolated per year. In France, serotype Typhimurium is the most prevalent serotype and remains stable with ≈4,000 human isolates per year; 1,4,[5],12:i:– strains have dramatically increased since 2005 (≈100 isolates) to reach ≈1,000 isolates in 2009.

We report a 2009 outbreak (8 cases) of salmonellosis in humans caused by a nonmotile strain of *Salmonella enterica* subsp. *enterica* with the antigenic formula 4,5,12:–:–. To estimate the extent of circulation and to determine the molecular subtypes of these nonmotile *Salmonella* strains, we performed a comprehensive, molecular epidemiologic study on human and nonhuman 1,4,[5],12:–:– strains isolated during 2000–2009 in France.

## The Study

In May 2009, diarrhea and fever developed in 8 persons living in southwestern France 1 day after they ate a homemade tiramisu prepared with raw eggs. Fecal analysis was performed on samples from 5 of the 8 persons. We also cultured a sample from the tiramisu. In medical laboratories, the isolation was performed by using standard procedures (i.e., use of conventional selective media). Isolation from the food sample was performed as required by the current International Organization for Standardization ISO 6579:2002 (i.e., by 2 selective enrichment media) (*9*). All cultures yielded *S. enterica* subsp. *enterica* 4,5,12:–:–.

An investigation at the suspected layer farm was conducted and showed the presence of 11 nonmotile *Salmonella* spp. isolates (with the same antigenic formula) in dust and feces collected from laying-hen houses. The layer farm, located in northwestern France, is a major farm that produces >32,000,000 eggs per year. All 17 isolates (5 from humans, 1 from the tiramisu, and 11 from the laying hens) were pan-susceptible to all antimicrobial drugs tested (*10*).

**Figure A1:**
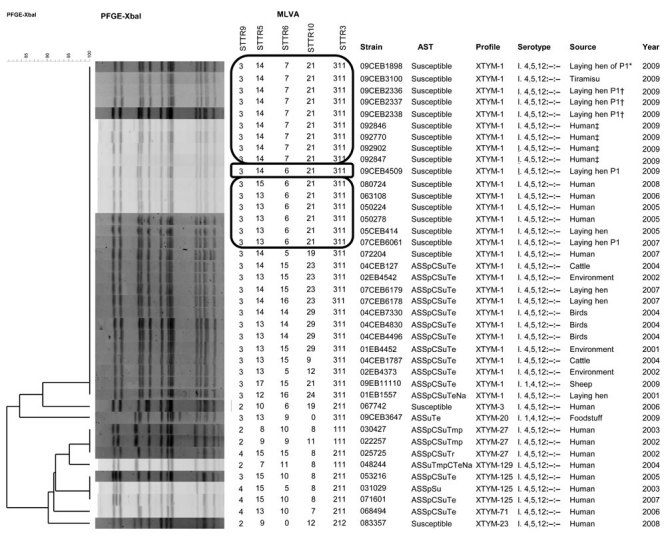
Phylogenetic analysis of *Xba*I–pulsed-field gel electrophoresis (PFGE) profiles obtained among a subset of 40 selected nonmotile *Salmonella enterica* serotype 1,4,[5],12:–:– strains isolated from humans and nonhumans during 2001–2009, France. Multilocus variable number tandem repeat analysis (MLVA), strain code, antimicrobial susceptibility testing (AST), PFGE profile found in our PFGE Typhimurium database, antigenic formula (serotype), and source and year of isolation are indicated to the right of the PFGE profiles. The MLVA groups indicated in the rectangles are the strains with the MLVA type found in our food poisoning investigation with its single- and double-locus variant with 1 repetition difference. P1 indicates the egg producer implicated in the tiramisu food poisoning. A, amoxicillin; S, streptomycin; Sp, spectinomycin; C, chloramphenicol; Su, sulfonamide; Te, tetracycline; Tmp, trimethoprim; Na, nalidixic acid. *Auto sampling; †authority sampling I; ‡tiramisu consumption; §authority sampling II.

Analysis by Pulsenet (www.cdc.gov/pulsenet/protocols.htm) standardized *Xba*I pulsed-field gel electrophoresis (PFGE) showed an indistinguishable profile, XTYM-1, associated with the multidrug-resistant *S. enterica* serotype Typhimurium DT104 clone (*10**,**11*) (Figure A1). Multilocus variable number tandem repeat analysis (MLVA) typing (*12*) showed an unique type 3–14–7–21–311 (loci STTR9–5–6–10–3, respectively) in 16/17 isolates and a single-locus variant (differing by 1 repetition) for the remaining isolate, which was an environmental isolate collected 1 month after the control measures were implemented (slaughter of laying hens) (Figure A1).

The tiramisu isolate 09CEB3100 was also characterized by multilocus sequence typing (MLST) for the *fliC* and *fljB* genes encoding the flagellar antigens (*13*). The isolate belonged to MLST sequence type 19, the main sequence type of serotype Typhimurium (http://mlst.ucc.ie/mlst/dbs/Senterica). PCR and sequencing identified the *fliC* gene encoding the i antigen and the *fljB* gene encoding for the 1,2 antigen, confirming that this *Salmonella* spp. strain of the antigenic formula 4,5,12:–:– was a nonmotile variant of serotype Typhimurium. The investigation and molecular data concluded that a nonmotile variant strain of serotype Typhimurium, genetically distinct from the emerging monophasic variant described worldwide, has been circulating in laying hens, whose contaminated eggs had likely caused food poisoning.

During 2000–2009, a total of 108,362 serotyped *Salmonella* spp. isolates from humans were registered at the FNRC-Salm, of which 374 (0.3%) were nonmotile. The 1,4,[5],12:–:– strains were the most prevalent (147/374, 39%). Such 1,4,[5],12:–:– strains have been rarely reported in food, environment or animals with 166 registered isolates collected by the French Food Safety Agency *Salmonella* network during 2001–2009 (compared with 21,214 serotype Typhimurium and 157,885 *Salmonella* spp. isolates registered).

To determine whether the population of nonmotile *Salmonella* spp. was circulating, we studied 43 additional *S. enterica* serotype 1,4,[5],12:–:– strains: 22 human and 21 nonhuman strains (5 from laying hens, 6 from cattle, 2 from sheep, 2 from partridges, 1 from chickens, 3 from the environment, and 3 from foodstuffs) isolated during 2001–2009. These strains were selected on the basis of their diversity (geographic area, year of isolation, and source). All the strains were characterized by antimicrobial drug susceptibility testing, PFGE, MLVA, and by *fliC* and *fljB* genes sequencing.

The 60 *S. enterica* serotype 1,4,[5],12:–:– isolates studied (17 isolates linked to the food poisoning and 43 additional isolates) displayed 22 different PFGE profiles. Only 10 profiles, from 49 isolates (81.6%), matched with profiles from the FNRC-Salm PFGE Typhimurium database (which includes 207 profiles generated in routine surveillance on 632 strains isolated during 1981–2010). All 49 strains were confirmed as nonmotile variants of Typhimurium by the identification of the *fliC* gene encoding the i antigen and the *fljB* gene encoding for the 1,2 antigen. The 11 strains whose PFGE pattern did not match any Typhimurium PFGE profile in our database belonged to serotypes Typhimurium (corresponding to 4 new PFGE profiles), Paratyphi B (n = 3), Agona, Derby, Indiana, and Saintpaul (1 each).

Among the 49 strains with a XTYM profile, 37 (75%) belonged to XTYM-1 (Figure A1). The nonmotile XTYM-1 strains were divided into 2 groups regarding their susceptibility to antimicrobial agents: pan-susceptible for those (17) linked to the food poisoning plus 6 strains isolated before 2009 (3 human and 3 nonhuman isolates) and penta-resistant profile (resistant to amoxicillin, streptomycin/spectinomycin, sulfonamide, chloramphenicol, and tetracycline) for the 14 remaining strains. Notably, all 23 nonmotile XTYM-1 pan-susceptible strains had a single amino acid substitution in the *fliC* gene (Asp251 encoding an asparagine residue) compared with those available in public database, including serotype Typhimurium reference strain LT2. The search was made with BLASTN (www.ncbi.nlm.nih.gov). The 12 nonmotile strains that matched other non–XTYM-1 profiles exhibited 7 PFGE profiles, suggesting the loss of motility was acquired independently by 7 distinct serotype Typhimurium populations.

Thirty-eight MLVA types were found after testing the 60 isolates. The MLVA type 3–14–7-21–311 found in the food poisoning strains was not observed in the other strains tested. A minimum-spanning tree based on the MLVA types is shown in the Figure. The strains from the food poisoning clustered at the external extremity of a tree branch, whereas 3 human and 3 laying hen strains (from the same producer) isolated earlier (2005–2007) clustered at the internal extremity of the same branch. The 2 MLVA types differed by only 1 repetition difference at 2 loci. Two laying-hen strains isolated in 2009 (also from the same producer) were grouped in the interconnecting node. This finding, combined with antimicrobial drug susceptibility testing, PFGE, and *fliC* sequencing data, suggested that the 2009 strain causing the food poisoning is a derivative of the 2005 strain; both strains were isolated from the same egg producer during a 4-year interval.

## Conclusions

Regarding the European Directive and the Commission Regulation on the monitoring and reduction of zoonotic agents (*14*), the French Regulation has extended the target for reduction of prevalence of *Salmonella* spp. in poultry producers to include notification of monophasic (because of the recent emergence in humans) and nonmotile (because of this food poisoning) variants of Typhimurium after January 2010 (*15*). This food poisoning outbreak also highlighted the need for a second selective enrichment media for *Salmonella* spp. detection not based on the motility in complement to the modified semisolid Rappaport-Vassiladis medium recommended as a single medium by the European Directive.

We report a foodborne outbreak caused by a nonmotile *S. enterica* 4,5,12:–:– strain in France. This strain has been present in laying hens in France for the past decade. Despite continuous advances in food safety and disease surveillance, control, and prevention, atypical pathogenic *Salmonella* spp. strains that bypass existing procedures do emerge. Foodborne bacterial infections remain a major public health concern.

## Figures and Tables

**Figure:**
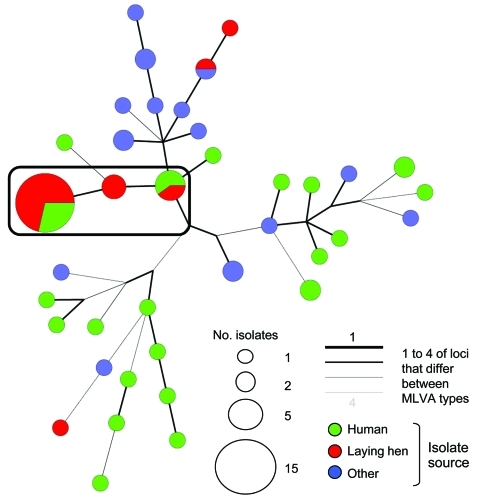
Minimum-spanning tree of multilocus variable number tandem repeat analysis (MLVA) of *Salmonella enterica* serotype 1,4,[5],12:–:– strains isolated from humans and nonhumans during 2001–2009, France. Each MLVA type is indicated by 1 node or branch tip, displayed as circles that are connected by branches of minimum-spanning tree. The length and the color of the branches represent genetic distances (changes in loci) between 2 neighboring types. The sizes of the different color circles depend on their population size. Wedges in circles indicate the proportion of isolates from respective sources with a particular MLVA type. A complex is shown in the black rectangle, based on maximum neighbor distance of changes at 2 loci and minimum size of 2 types. This specific complex linked the tiramisu food poisoning strains and other loci-derived strains for 2005–2009.
